# Buckypaper made with carbon nanotubes derived from CO_2_†

**DOI:** 10.1039/d4ra04358h

**Published:** 2024-08-27

**Authors:** Gad Licht, Kyle Hofstetter, Stuart Licht

**Affiliations:** a Direct Air Capture LLC, A4 188 Triple Diamond Blvd North Venice FL 34275 USA slicht@gwu.edu; b Carbon Corp 1035 26 St NE Calgary AB T2A 6K8 Canada; c Dept. of Chemistry, George Washington University Washington DC 20052 USA

## Abstract

Unusual buckypapers, sheets of graphene nanocarbons (GNCs) such as carbon nanotubes, were formed with GNCs directly derived from CO_2_*via* molten carbonate electrolysis. Examples are presented for buckypapers made from CO_2_ using either crushed or chemically washed GNCs, epoxy-infused CNTs, or GNCs pressed directly using a hot electrolyte to remove excess electrolyte.

## Abstract 2

A carbanogel made from CO_2_ was used to make new forms of buckypaper.

## Graphical abstract

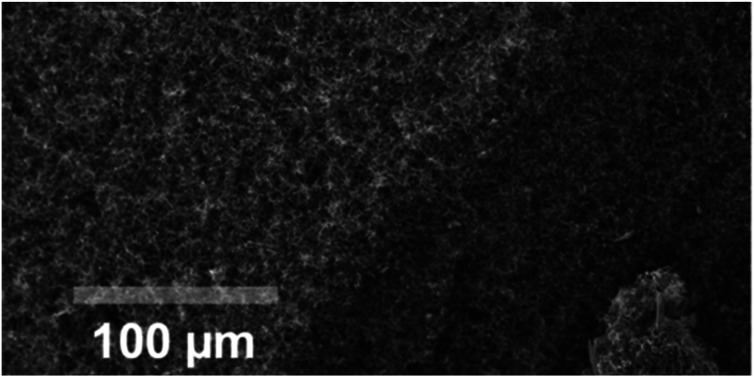

## Introduction

Buckypapers are graphene carbon nanocarbon (GNC) sheets. The majority of studies report on buckypapers are formed from the GNC consisting of carbon nanotubes. Additionally, buckypapers of graphene, graphene oxide, and carbon nano-onions have been studied. Buckypapers have demonstrated enhanced physical and chemical properties, including but not limited to high tensile strength; high electrical conductivity; high thermal conductivity; electronic shielding; magnetic shielding; electrical charge storage for use in batteries, fuel cells, and capacitors; piezo resistivity; catalytic activity; reduced friction; and targeted therapeutic activity.^1–15^ Many of these properties originate from planar sp^2^ bonded carbons of graphene in different geometric arrangements within a buckypaper GNC sheet. Sheet properties can be modified through surface or covalent functionalization, morphology modulation, and doping.^16–21^

Buckypapers are often integrated within a composite to enhance the physical, chemical, or electrical properties of the buckypaper-free material.^22–26^ Buckypapers are attractive as they are lower in weight, stronger, more conductive, and more resilient for applications such as strain, fracture, chemical and motion sensors;^13,27–36^ electrochemical reactors^37^ for water treatment;^4,37,38^ EMF shielding/absorbent coatings;^7–9^ batteries and supercapacitors;^15,17,18,23,24^ stronger plastic composites such as aeronautics^11,12,25^ and medicine.^39,40^

Despite the extensive academic interest and wide range of applications, the acceptance and widespread use of buckypaper has been hampered by the high cost and carbon-footprint of graphene carbon nanocarbon synthesis. The manufacturing process generally includes a chemical vapor deposition (CVD) process used in the commercial production of carbon nanotubes, graphene, and carbon nano-onions. GNCs have shown promise, but their CVD production cost has remained prohibitively high in commercial settings, with the resulting products carrying both a significant cost and a significant carbon footprint.^41^ Currently, the price of GNCs such as CNTs, graphene, and carbon nano-onions are in the range of USD $100,000–$10 million per tonne. Comparatively, steel is priced at $400 to $700 per tonne.

To form buckypapers, CVD-formed GNCs are often first added to a liquid, then sonicated to provide a homogenous dispersion, and the liquid is filtered and/or dried off, leaving the buckypaper formed as a solid sheet of dispersed GNCs.

The years 2009 and 2010 marked significant milestones in the exploration of splitting CO_2_ into carbon (C) and oxygen (O_2_) through molten carbonate electrolysis, offering a decarbonization path to combat climate change. Building upon this progress, research in 2015 revealed that transition metal nucleus growth during this electrolysis process facilitates the direct conversion of CO_2_ into pure CNTs and other GNCs.^42,60^

1 CO_3_^2−^ (molten) +/− 4e^−^ → C (GNC) + O_2_ (gas) + O^2−^ (dissolved)

CO_2_ undergoes a chemical reaction with electrolytic oxide, as depicted in eqn (1), to regenerate CO_3_^2−^ according to eqn (2).

2 CO_2_ (gas) + O^2−^ (dissolved) → CO_3_^2−^ (molten)

The integration of eqn (1) and (2) results in the following net reaction:

3 CO_2_ (gas) → C (GNC) + O_2_ (gas)

Various GNCs have been synthesized, including helical, thin-walled, magnetic, and doped CNTs with carbon nano-bamboo, nano-pearl, and nano-tree morphologies as well as single-layered or multilayered graphene (nano-platelets), hollow or concentric buckyball spheres (nano-onions), and three-dimensional structures such as graphene nano-scaffolds.^44–55^ Manipulating the electrolysis parameters, such as temperature and current density, allows for the tailored production of specific GNCs. For instance, lower temperatures of about 725 °C are typical for forming carbon nano-onions,^44^ while a higher range from 750 to 770 °C is utilized for CNT syntheses.^45–47,49,52–58^

This communication presents a new low-cost process for the synthesis of graphene nanocarbons sheets (GNC buckypaper). CO_2_ is the sole chemical reactant in eqn (3), and 4 tonnes of CO_2_ are consumed for each tonne of C_(GNC)_ formed. Carbanogels are GNC lattices formed by carbon capture directly through the electrolytic splitting of carbon dioxide (CO_2_). The electrolysis occurs in molten carbonate. The reduced CO_2_ product builds up as a carbanogel containing the GNC and excess electrolyte at the cathode. The carbanogel product is recovered hot (as a red-hot slush) or cold (as a solid) by scraping and/or pressing from the cathode. Pressing returns excess electrolyte to the electrolysis chamber. Residual electrolyte and impurities may be removed with thermal, mechanical, or electrochemical treatment. The carbanogel is crushed and compressed within a mold to form the buckypaper product.

Herein, buckypaper was composed of GNCs derived from CO_2_. This buckypaper presents an opportunity to utilize the greenhouse gas CO_2_ for producing stable GNCs, contributing as a reliable decarbonization methodology for the long-term removal of CO_2_. Graphite, a macroscopic form of layered graphene, serves as a geological stability benchmark with a lifespan spanning hundreds of millions of years, providing a stability reference for GNC materials.

## Results and discussion

### Design of the experiment and carbanogel formation

Lithium carbonate was purchased at a battery grade >99.5% and was used as received. As analyzed, the lithium carbonate had a composition of 99.8% (Li_2_CO_3_, Shanghai Seasongreen Chemical Co.). Muntz brass is a high-zinc brass alloy composed of 60% copper and 40% zinc; this material is also referred to as 280 brass. This material serves as the cathode and was purchased from https://onlinemetals.com/ and in larger quantities from Marmetal Industries. Electrolysis was conducted in 304 stainless steel “carbon pots”. The pot acts as both the cell case and its inner walls serve as the anode.

In a 770 °C molten Li_2_CO_3_ environment, CO_2_ underwent splitting according to eqn (1)–(3), utilizing a Muntz brass cathode and a 304 stainless steel anode at a constant electrolysis current density *J* of 0.4 A cm^−2^. Throughout the electrolysis process, CO_2_ was divided into O_2_ and GNCs. The GNCs developed as a network of interconnected graphene nanocarbons and electrolyte on the cathode. An expanded description of this electrolysis procedure, including product separation from excess electrolyte and product washing, has been recently delineated.^56^ The mixture of GNCs and carbonate electrolyte is referred to as a carbanogel and further refined through the electrolyte's separation process, as detailed in the ESI.†,^56,57^ Thermogravimetric analysis (TGA) of the product, conducted using a PerkinElmer STA 6000 TGA/DSC, revealed a purity exceeding 97% (with less than 3% residual oxidation impurities at 800 °C) and exhibited an inflection temperature of 608 °C, indicating remarkable resistance to oxidative combustion, characteristic of graphene-like materials. Scanning electron microscopy was studied using a PHENOM Pro-X scanning electron microscope to examine the products of the electrolysis process at various magnifications.

**Fig. 1:**
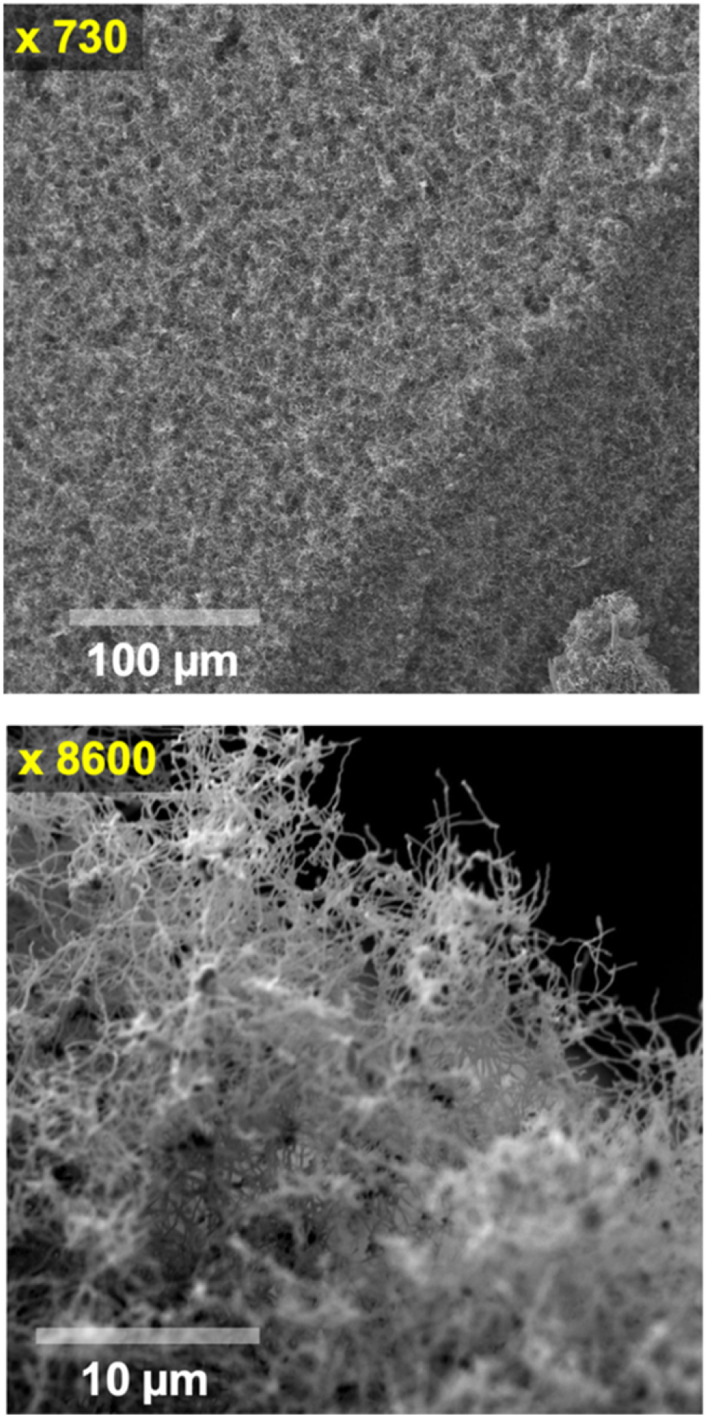
Medium (top) and high (bottom) scanning electron microscopy images of the carbanogel particles comprised of carbon nanotubes formed at 770 °C Li_2_CO_3_ at *J* = 0.4 A cm^−2^.

Fig. 1 presents a carbanogel with reduced electrolyte content achieved through a concentrated HCl wash. In a later example, the carbanogel was instead directly pressed to remove residual electrolyte. In Fig. 1, the 770 °C molten Li_2_CO_3_ electrolyzed sample was observed at two different SEM magnifications (x720 and x8600). This specific example illustrates the CNT carbanogel synthesized through CO_2_ electrolysis, synthesized in 770 °C molten Li_2_CO_3_ detached from the cooled cathode, fragment, acid washed, and utilized in the first several buckypaper GNCs. Due to its lower combustion temperature compared to GNCs, amorphous carbon is more susceptible to oxidation, and both amorphous carbon, residual electrolyte, and metal impurities can be removed by oxidation and/or washing, as confirmed by electron dispersive spectroscopy (EDS) and TGA. Useful alternative post-electrolysis product washes include formic acid, copious water, or ammonium sulfate, although the latter two primarily remove excess electrolyte without affecting the amorphous carbon or metal impurities. Another alternative wash, combining hydrochloric acid and hydrogen peroxide, eliminates excess electrolyte, metal impurities, and amorphous carbon impurities, particularly coupled with sonication during the product wash.

The top SEM image in Fig. 1 presents a large collective size of the intertwined CNTs forming the carbanogel. One advantage of grouping nanoparticles within an intertwined macroscopic matrix is the mitigation of respiratory hazards typically associated with nanoscale particles during transportation.

Higher-resolution SEM image (Fig. 1 bottom) reveals that the GNC product comprises highly pure carbon nanotubes. The SEM shows the diverse lattice intertwined orientation of the carbanogel product. This product is utilized in subsequent buckypaper formation steps. Additionally, this structure offers electrical and thermal conductivity pathways, along with a highly porous framework suitable for accommodating polymers, catalysts, or battery intercalation. We have recently reported extensive SEM, TEM, X-ray, Raman, high angle annular dark-field (HAADF) elemental analysis/TEM of the CNT and various GNC products, as detailed in the ESI.†,^51,58^

**Fig. 2:**
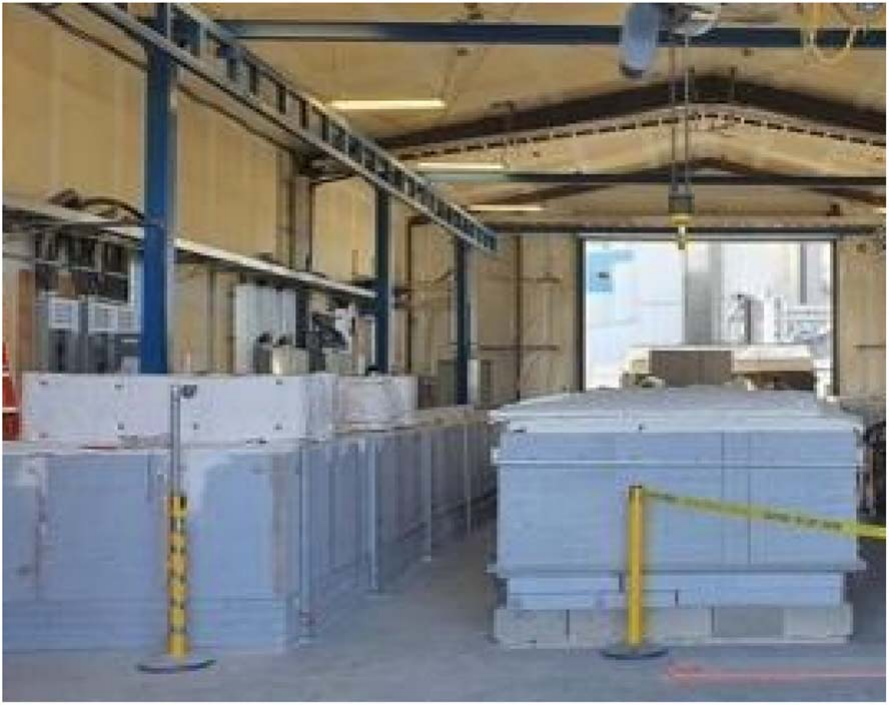
The Genesis Device® kiln modules facilitate large-scale CO_2_ molten carbonate electrolysis and was used in this study. Carbon Corp operates these modules in Calgary, Canada, for onsite decarbonization.

For the electrolytic CO_2_ splitting and transformation electrolyses of this study, the kilns utilize a direct (untreated) feed of 5% CO_2_ from the emissions of the adjacent 860 MW (Shepard, Calgary Canada) natural gas electric power plant or direct air. The large kiln modules designed and utilized are pictured in Fig. 2. These kilns simultaneously sustain electrolysis in several carbon pots. Cathodes show an increase in the surface area to over 10 000 cm^2^. In each case, the cathode is mounted vertically in the electrolyte, across from which are the 304 stainless steel anodes; the anodes simultaneously function as the electrolysis anodes and as the chamber walls of the 304 stainless steel “carbon pot”. The electrolyte has a strong affinity for CO_2_ from the open air. The kilns shown in Fig. 2 can also be configured for direct air use. Finally, an amine concentrator is also in place at the site that can concentrate the 5% CO_2_ flue gas. From the amine concentrator as an alternative source, 98% CO_2_ (containing 2% H_2_O) was used as the kiln/carbon pot input, producing equivalent GNCs.

### CO_2_ buckypaper (BP) with CNTs

**Fig. 3:**
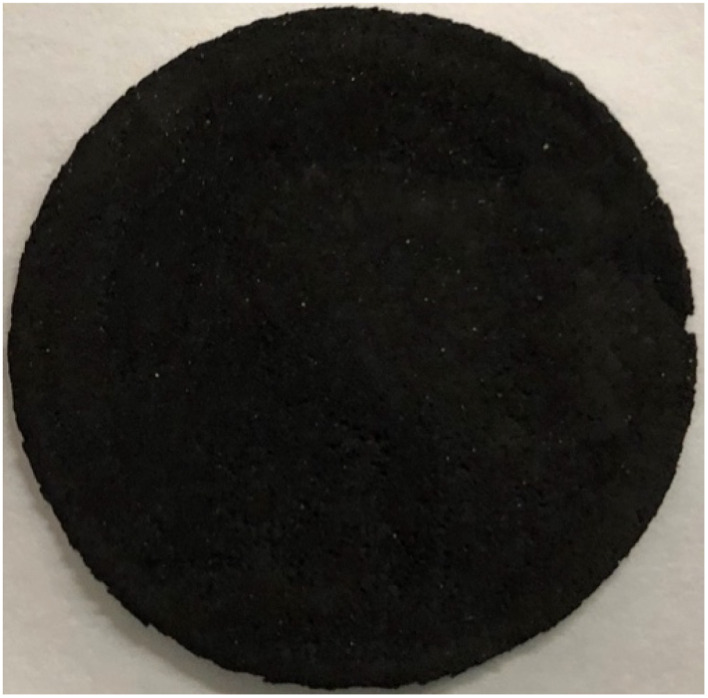
A 4.7 cm diameter carbon nanotube buckypaper prepared *via* the electrolysis of CO_2_ to form a carbanogel, grinding of the product, dispersion in isopropanol, pressing and filtering, and forming on a 0.2 μm pore nylon membrane.

Fig. 3 presents a typical example of a GNC buckypaper produced from CO_2_. In this initial example, the GNC buckypaper was generated through electrolysis to convert CO_2_ into carbanogel. The carbanogel product was cooled, removed from the electrode, ground, washed, dispersed, and molded to CNT buckypaper. Specifically, the process involved utilizing a 304 stainless steel case within a 770 °C Li_2_CO_3_ molten electrolyte, employing a Muntz brass cathode and a 304 stainless steel anode to yield the CNT carbanogel product (Fig. 1). Subsequently, this carbanogel product underwent cleaning with hydrochloric acid (HCl) and 0.2 grams of the washed product was mixed in 300 mL of isopropyl alcohol, followed by sonication for 30 minutes to ensure even dispersion.

The dispersed isopropanol CNT mixture was then transferred into a vacuum filter assembly (Whatman nylon membrane filter; 0.2 μm Pore, 47 mm diameter), pressed, the liquid was extracted under vacuum, dried, pressed, and detached by lifting from the membrane filter. The GNC buckypaper had a thickness of 96 μm, and a measured conductivity of 11 000 S m^−1^ (*σ* = length/(*R*, resistance, *x* cross section)). This conductivity is consistent with our measurements of carbonate-synthesized CNTs and may be increased an order of magnitude by boron doping (addition of a boron salt during the electrolysis).^52,53^

### Composite, larger, and improved dispersion BP from CO_2_

Buckypaper strengtheners can include, but are not limited to epoxies, resins and other polymers, cementitious materials, and metals. Catalysts to expedite chemical or electrochemical reactions can also be included. Dopants to affect the physicochemical properties of the BP can be added. Effective GNC dopants can enhance the conductivity, catalytic activity, and battery storage capacity properties. Dopants include boron, nitrogen, sulfur, and phosphorous.^52–54^ A magnetic material is one or more of those with ferromagnetic properties, paramagnetic properties, diamagnetic properties, and any combination thereof.^4,46^

Jetset-Metlab epoxy is a rapidly cured BPA epoxy. Its resin comprises 80–90 wt% propane, 2,2-bis[*p*-(2.3-epoxypropoxy)phenol] polymers, and 10–20 wt% alkyl (C12–14) glycidyl ether. The hardener consists of 60–70 wt% diethylenetriamine, 30–40 wt% bisphenol A, less than 0.8% aminoethylpiperazine, and less than 0.2% ethylenediamine. In a manner similar to the buckypaper shown in Fig. 3, buckypaper (BP) was formed from 0.25 g of the carbanogel. However, the buckypaper was not pressed, resulting in a considerably thicker (1133 μm) BP with an order of magnitude lower density (0.2 g cm^−3^). 3.6 g of the Jetset-Metlab epoxy was mixed with 0.38 g of the Jetset-Metlab hardener. 0.38 g of the epoxy mix was spread on the BP. A silicone sheet was placed over the epoxy-BP, compressed with a heavy (0.5′′ thick) stainless steel plate, and cured overnight. The silicone sheet was readily removed and the epoxy was infused into the BP and hardened. The composite, again formed with CNT from CO_2_, was readily handled and highly resistant to fracturing or breakage. In an upcoming paper, we will present that compared to samples without CNTs, even low levels of added CNTs (1.5 to 2 wt% CNT) increase the tensile strength of several epoxies. Specifically, Timber Cast, Varathane and Jetset-Metlab epoxies' tensile strengths were increased by 40 to 60% in ASTM D638 “dogbone” type V mold samples tested with an ETM-10 kN Computer Controlled Electronic Universal Testing Production Machine (Shore D Durometer). The hardness was also increased. Equivalent tests on the BP resin composite, upon completion, will be reported in an expanded paper.

The buckypaper from CO_2_ presented in Fig. 3 is scalable. However, thinner BPs, consisting of BP formation with a decrease in the grams of carbanogel per unit area of the membrane, were increasingly difficult to detach from the filter paper. Intermediate diameter BPs could be formed and removed. Specifically, when 0.3 g of ground carbanogel was washed in HCl, dispersed by sonication for 30 minutes in isopropanol, and vacuum filtered through a 270 mm Whatman 0.2 μm pore (grade 4) nylon filter, the BP was formed but could not be removed in one piece from the filter paper.

**Fig. 4:**
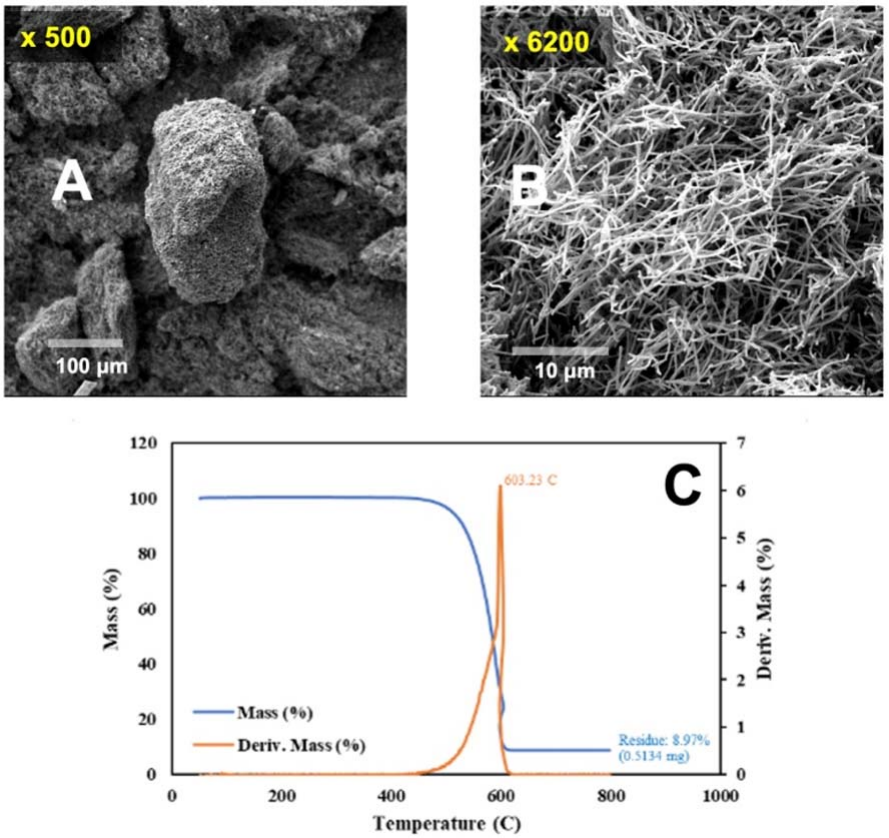
(A) Medium and (B) higher magnification scanning electron microscopy images of the carbanogel particles composed of carbon nanotubes. (C) TGA of the carbanogel product, formed in 750 °C Li_2_CO_3_ at *J* = 0.09 A cm^−2^.

A modified dispersion method to facilitate BP detachment was sought. Polyvinylpyrrolidone (PVP) was utilized to promote the dispersion of CNTs.^59^ 0.05 g of 10 000 molecular weight PVP (PVP10, from Sigma Aldrich) was added along with 0.10 g of CNTs, rather than 0.20 g, ground from the washed CNT, and dispersed in 300 mL of isopropanol with 15 minutes, rather than 30 minutes, of sonication. The CNTs were prepared in a manner similar to those presented in Fig. 1, also in Li_2_CO_3_, but at a lower temperature of 750 °C and a lower current density of 0.09 A cm^−2^. Further, the carbanogels were more extensively ground using a Magic Bullet Blender. The CNT's SEM and TGA are presented in Fig. 4.

**Fig. 5:**
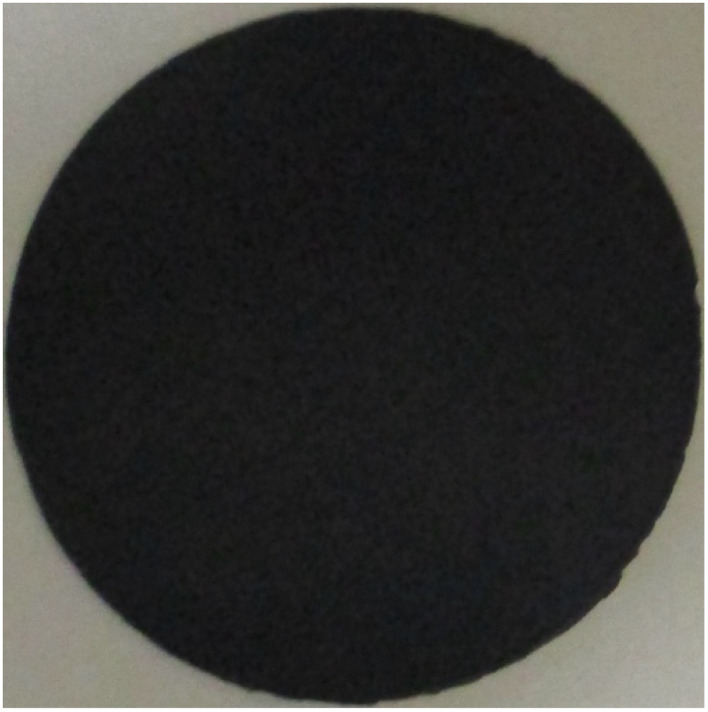
A 4.7 cm diameter carbon nanotube buckypaper prepared *via* the electrolysis of CO_2_ to form a carbanogel, grinding of 0.10 g of the product, dispersion with PVP in isopropanol, pressing, filtering, forming on the 0.2 μm pore nylon membrane, and lifting from the membrane.

Once again, in Fig. 4, the carbanogel particles are evident in the lower magnification SEM (panel A), and the TGA temperature of inflection of 603 °C is high, although the oxidized 9% residual (panel D) is higher than that in the previous sample. Fig. 5 presents a typical example of a GNC buckypaper produced with the PVP isopropanol-dispersed CNT from CO_2_. The unpressed BP is considerably thinner at 58 μm than the unpressed sample prepared without the PVP dispersant.

### Direct press CO_2_ buckypaper

The electrolyte has a strong affinity for CO_2_, directly from air (pCO_2_ = 420 ppm and climbing), or directly sourced from an industrial stack. Industrial CO_2_ feedstocks tested vary from pCO_2_ = 50 000 ppm (from the Shepard Energy Centre 860 MW natural gas power plant, Calgary, Canada) to pCO_2_ = 980 000 ppm (from a liquid amine concentrator onsite at Alberta Carbon Capture Technology Centre, ACCTC, at the plant). The electrolysis provides a continuous source of oxide (eqn (1)), reacting with CO_2_ (eqn (2)). The capture and transformation of CO_2_ was determined by ^13^C isotopic labeling,^60^ and the real-time simultaneous measurement of CO_2_ and the eqn (1) co-product O_2_ was done as presented previously.^55^ In the first examples, buckypapers were formed with carbanogels by the transformation of CO_2_ in the air. In this next example, the BP was formed from 50 000 ppm CO_2_ fed from the Shepard Energy Centre natural gas power plant exhaust.

**Fig. 6:**
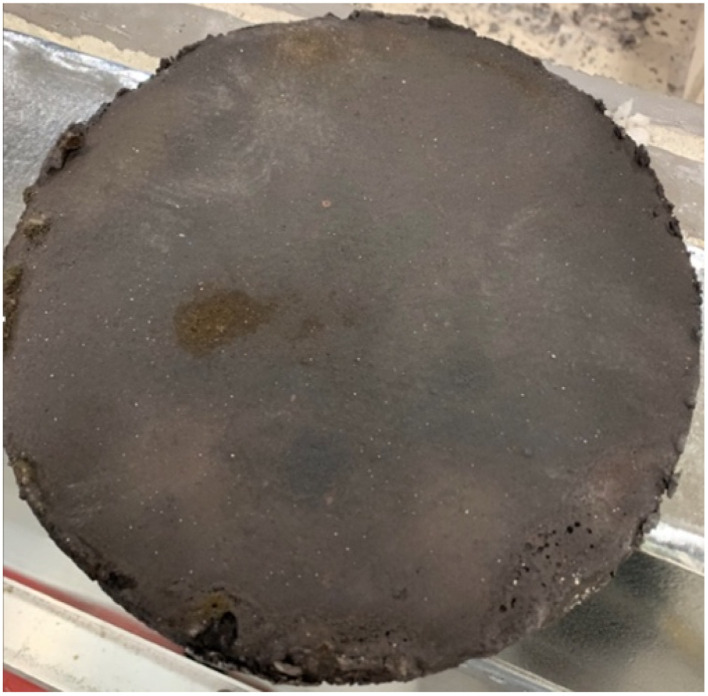
A 35 cm diameter carbon nanotube buckypaper prepared *via* the electrolysis of CO_2_ to form a carbanogel, and pressing of the carbanogel product while molten to separate residual carbanogel electrolyte from the graphene nanocarbon, carbon nanotube, and product.

Fig. 6 presents an alternate methodology for the preparation of a GNC buckypaper derived from CO_2_. This GNC buckypaper was produced from CO_2_ transformed into carbanogel, following the process outlined in the previous example. However, instead of chemical washing, the electrolyte content of the carbanogel was reduced through direct compression while the carbanogel was hot (containing the GNC product and residual, excess electrolyte). Specifically, the carbanogel, containing the solid GNC and molten electrolyte, was generated at the cathode and then compressed at 500 psi through layers of 60 mesh 304 alloy stainless steel mesh while hot, as recently delineated.^56^ Notably, the 250 μm mesh pore size is significantly larger than that of GNC but smaller than that of the carbanogel particle size. The small area of the lighter brown discoloration in Fig. 6 is where the GNC buckypaper adhered to the mesh screen during separation.

During compression, the larger size of the intertwined CNTs in the carbanogel is retained above the mesh, while the electrolyte passes through. The resulting GNC buckypaper's thickness is linearly proportional to the starting mass of the carbanogel and approximately inversely proportional to the applied pressure.

The GNC buckypaper shown in Fig. 6 has a diameter of 350 mm, and larger versions, approximately two-fold in size, were produced. A 3700 psi example is seen in our recent carbanogel electrolyte extraction study.^56^ Unlike other buckypaper formation protocols, which call for a prerequisite dispersion step such as sonication, to provide a homogeneous distribution of the GNC components, the GNC components are already homogenously distributed in the unground carbanogel. Hence, the BP formation process does not need a prerequisite GNC dispersion.

### CO_2_ BP generalization and future potential

A broad array of filter pore sizes and solvents are found to be effective for forming buckypaper from CO_2_ transformed into the carbanogel using these BP formation methodologies. Varying the CO_2_ electrolysis parameters, including temperature, current density, and cathode, anode, and electrolyte composition, generates alternative GNCs for buckypaper (such as CNOs,^44^ to be described for buckpaper in a subsequent study).

Forces to align, rather than disperse, the CNMs can additionally be applied during the carbanogel buckypaper formation and or liquids added before the pressing to maintain more even layering of the carbanogel particles. The alignment can provide directional, anisotropic properties to the carbanogel buckypaper and provide enhanced carbanogel buckypaper properties, including but not limited to enhanced strength, conductivity, and directional interactions with visible and other electromagnetic radiation. The applied alignment forces can be linear, radial, cylindrical, or spherical to produce directional geometries of anisotropy.^23,60–64^

The GNC components in the BP can be aligned mechanically, electrically, or magnetically during the BP formation or a combination thereof to further enhance the BP properties, including enhancements of the BP strength and/or electrical and thermal conductivities and EMF absorption, and will be presented in an expanded article. The mechanical alignment can be achieved with the application of shear force, such as by pulling or spinning during the BP preparation steps or dragging a piston applying the formation pressure. Alternatively, the shear force can be directionally applied to increase rather than align the GNC entanglement. The electrical and/or magnet alignment is achieved with the application of an orienting electrical or magnetic field during the BP preparation or processing stages. Magnetic GNCs are prepared to incorporate magnetic materials, such as metals or metal carbides, during the GNCs preparation stage. The decrease in the distance of the magnetic field is greater than that for the electric field, and the magnetic alignment is more offset than the electric alignment effect by the competing random disorder of Brownian motion, which increases with temperature and modes of freedom and decreases with increasing molecular mass and viscosity. Hence, the alignment is enhanced by the decrease in temperature or molecular mass (as exemplified by multiwalled *versus* single-walled carbon nanotubes) and increases with viscosity.

The carbanogel buckypaper may be used alone, such as in liners, heat retardants, or shields, or in combination, such as laminates, with other materials to impart improved properties to other materials. CNT materials have displayed a shape-memory property (spontaneously returning to their original shape) under thermal, mechanical, electrical, or magnetic activation conditions which can be incorporated into BP.^65–74^ This shape-memory effect is promoted by anisotropic properties in the buckypaper. The electrical and thermal conductivity of buckypaper present superior properties for their applications as heating elements. Both shape-memory and heating element behavior have been studied alone and in combination with other material layers.^68^

### Incentivized carbon mitigation

Innovative approaches, such as employing high-solubility molten pathways and harnessing renewable energy for electrolysis, have shown promise in reducing energy consumption and production costs. In the formation of graphene nanocarbon composites (GNCs) *via* CO_2_ to carbon nanoallotrope technology (C2CNT®), the process solely requires CO_2_ as a reactant. The energy required for electrolysis to convert CO_2_ into GNCs falls within the range from 0.8 to 2 volts.^60^ When produced in bulk, they are approximately $1000 per tonne.^53^ These are akin to the costs of the industrial aluminum production electrolytic process, which involves splitting aluminum oxide to yield commercial-grade aluminum metal.^53^ C2CNT® costs will be further reduced as solar and wind energy are increasingly used as alternative power sources.^75–84^

An estimate of cost and decarbonization benefits is exemplified in the context of one application. Typical ratios of plastic resin to fiber infusion are in the range of 60 : 40. The price index for plastic materials and resins has been $300–$400 per tonne over the last three years.^85^ Hence, the infusion of resin in buckypaper may decrease the material costs to the order of $500 per ton buckypaper resin composite while significantly lowering the quantities of the material to achieve the properties needed for various applications. The buckypaper-resin composite provides the capability of enhancing the strength, tearing resistance, and fracture toughness several folds while increasing the electrical and thermal conductivity by several orders of magnitude. Reducing the amount of plastic needed to achieve the desired properties through buckypaper resins made with GNCs from CO_2_ provides the incentive of both cost and carbon footprint reduction.

## Conclusions

Buckypapers with GNCs, such as carbon nanotubes made from CO_2_, are demonstrated to form a range of conductive, liquid-dispersed and unusual direct molten pressed forms of buckypapers, all prepared from lattices of intertwined carbon nanotube carbanogels synthesized by the electrolytic splitting of CO_2_ in molten carbonates. As with industrial aluminum production by the electrolytic splitting of aluminum oxide, the production of GNCs by the electrolytic splitting of carbon dioxide is inexpensive, providing a cost-effective path for the synthesis of buckypapers. The only reactant used in the production of the GNCs is CO_2_ (3.7 tonnes of CO_2_ are consumed for each tonne of C_(GNC)_ formed), providing a decarbonization path to mitigate the greenhouse gas CO_2_. The captured carbon, transformed from CO_2_, provides a storage buffer to remove CO_2_. Furthermore, the removed CO_2_ in the form of GNCs is highly stable, with the potential to permanently sequester CO_2_ from the carbon cycle. It is anticipated that with the further alignment and shape-memory effects that can be achieved with buckypapers, a range of useful lightweight, high strength, and high conductivity applications can be demonstrated with these new materials.

## Data availability

The data supporting this article have been included as part of the ESI.†

## Conflicts of interest

There are no conflicts to declare.

## Supplementary Material

RA-014-D4RA04358H-s001
