# Supplementary material for: Buckypaper made with carbon nanotubes derived from CO2
Source: RSC Adv. 2024 Aug 27;14(37):27187–95. doi: 10.1039/d4ra04358h (PMC11348762; doi:10.1039/d4ra04358h)
Supplement: RA-014-D4RA04358H-s001 [file RA-014-D4RA04358H-s001.pdf]

Supplementary Information (SI) for RSC Advances.
This journal is © The Royal Society of Chemistry 2024

Supplementary Information accompanying:

Buckypaper made with Carbon Nanotubes made from CO_2

Gad Licht, Kyle Hofstetter and Stuart Licht
George Washington University, Washington, DC, USA,
Carbon Corp, Calgary, Canada & Direct Air Capture LLC, Florida, USA

Elevated levels of atmospheric CO_2 are the primary driver of global warming. Historically, atmospheric CO_2 concentration fluctuated within a range of 235 ± ~50 ppm over the past several hundred thousand years, until 1850. Currently, it stands at 426 ppm and continues to increase annually, leading to widespread climate disturbances, habitat degradation, and species extinction [1-4]. The inherent chemical stability of CO_2 poses a significant challenge to its removal by conversion into a non-greenhouse material, a subject explored in our US NSF workshop on Chemical Recycling and Utilization of CO_2 [5]. However, overcoming this stability of CO_2 and utilizing it as a carbon-negative precursor for the production of valuable products provides an incentive for the reduction of this greenhouse gas.

In 2015, it was shown that the growth of transition metal nuclei during this electrolysis process leads directly to the conversion of CO_2 into pure graphene nanocarbons, including carbon nanofibers and carbon nanotubes (CNTs) [6]. This transformation of the greenhouse gas CO_2 into valuable GNC products offers a chance to convert CO_2 into a form of carbon stabilized by graphene, thus aiding in mitigating climate change. Graphite is an analogous macroscopic form of layered graphene, and as a mineral graphite has an established geologic (hundreds of millions of years) lifetime.

The CO_2 to nanocarbon process, including electrolyte separation and return to the electrolysis chamber, and extraction of the pure GNC product is illustrated Figure S1. As illustrated in Figure 1, during electrolysis CO_2 either sourced directly from the air or industrial emissions are transformed to GNCs by electrolysis in molten carbonates. The CO_2 is split into C and O_2 with a GNC-electrolyte matrix growing at the electrolysis cathode. This

nanocarbon/carbonate electrolyte mix has been termed a carbanogel and is refined through the separation of the electrolyte.

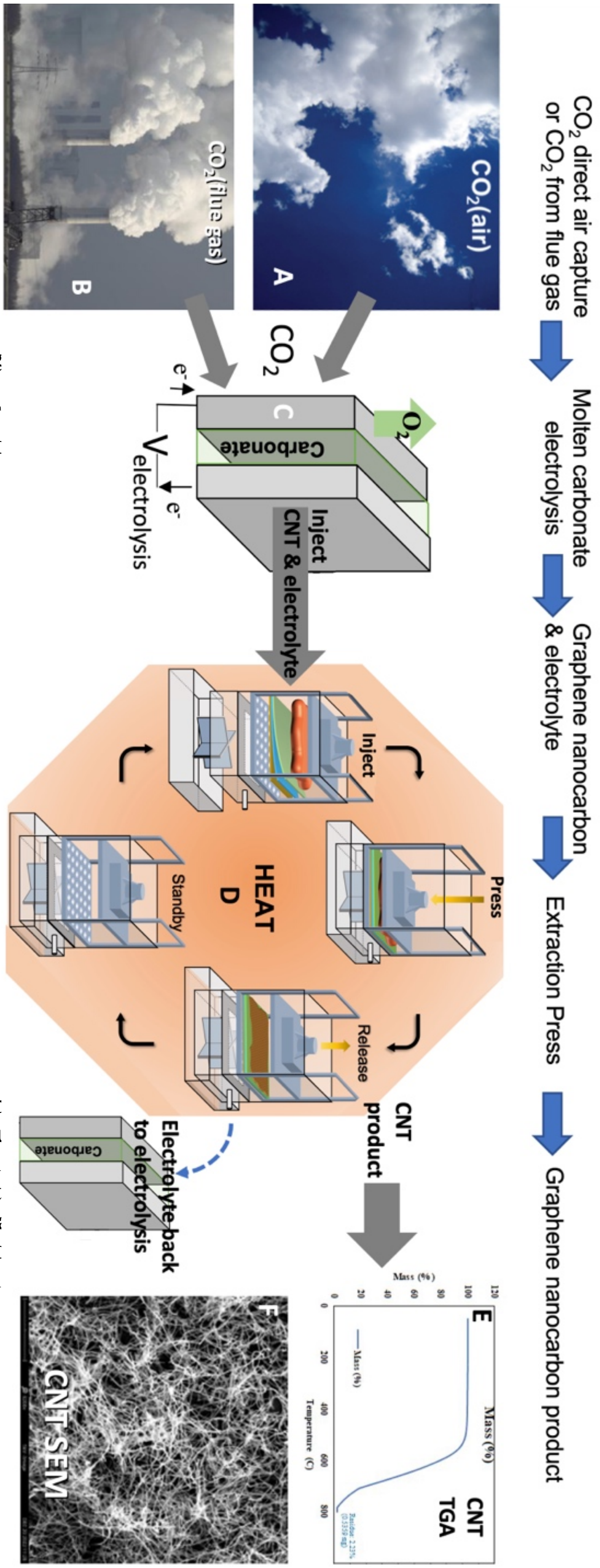

1 Figure S1. The CO_2 to g[illegible] an anthropogenic source (CCUS) or from [illegible] into graphene nanocarbons, panel F. T[illegible]ned by tuning the electrochemical conditi[illegible] The nanocarbon product (carbon nanotub[illegible]e by a high-pressure, high-temperatu[illegible] morphology, as indicated by SEM panel [illegible]ure oxidation as characterized. With part[illegible]G. Licht and S. Licht, Green and scalable separa[illegible]by efficient high-temperature press filtratio[illegible] 10.1016/j.seppur.2020.11[illegible]

.

The high electrical conductivity character of the graphene nano-allotropes supports continuous growth during the CO_2 molten electrolysis at low electrolysis voltage. This cathode product grows as an interconnected matrix with electrolyte in the matrix pores. This matrix containing carbonate electrolyte has been termed a carbanogel. Some of the electrolyte in this matrix is rather loosely bound. For example, a post-electrolysis cathode lifted out of the molten electrolyte can release over 30% of the bound electrolyte by gravitational drip.

Figure S2 illustrates larger vertical presses that have been scaled up, which include the transfer of applied pressure to the pressing chamber using a hydraulic ram, as described previously[9]. In Figure S2A, there's a cross-sectional depiction of an intermediate scaled-up carbanogel electrolyte extraction unit, detailing the plunger, filter screen platform, and electrolyte exit chamber [9]. Figure S2B shows a larger carbanogel electrolyte extraction unit in operation, capable of pressing up to 0.25 tonnes of carbanogel. Presses in the unit with 50 kg carbanogel have already achieved over 99% electrolyte extraction efficiency.

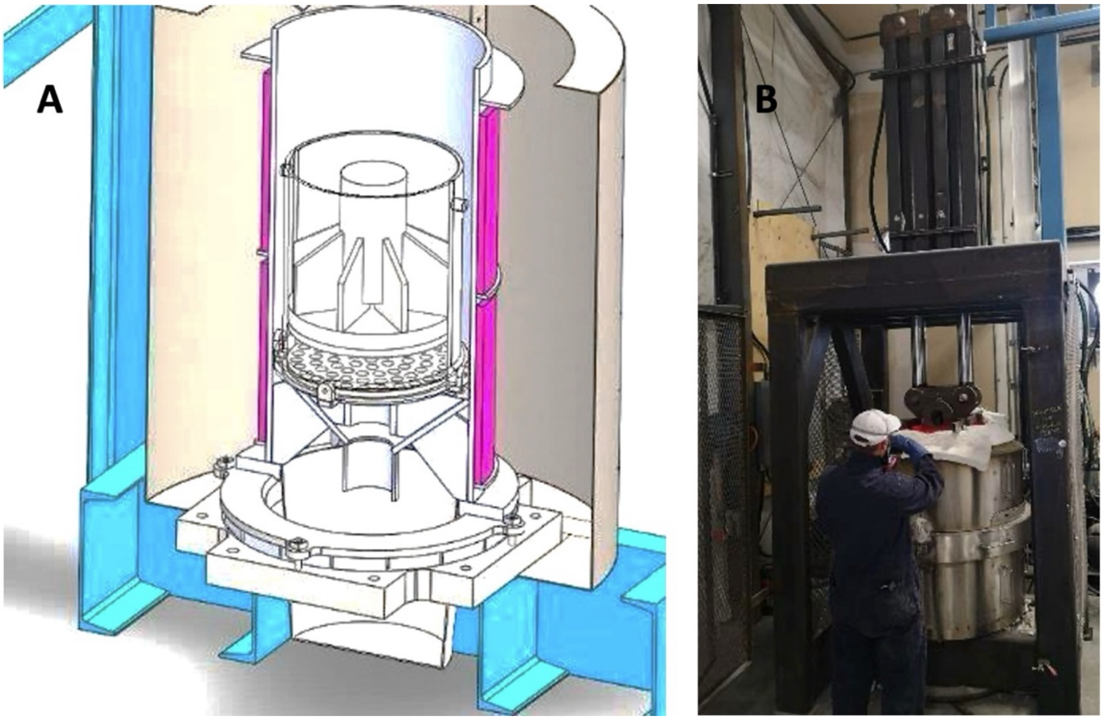

Figure S2. Scaled-up carbanogel electrolyte extraction units. A) Cross-sectional illustration of a mid-scaled extraction unit illustrating the plunger, the filter screen platform and the electrolyte exit chamber. B) Larger scaled carbanogel electrolyte extraction unit in action. From open access paper Licht, K. Hofstetter, S. Licht, Separation of molten electrolyte from the graphene nanocarbon product subsequent to electrolytic CO_2 capture. Decarbon 4 (2024) 100044. https://doi.org/10.1016/j.decarb.2024.100044.

Control of the electrode and electrolyte composition, and CO_2 electrolysis splitting temperature and current density tunes the decarbonization process to form a range of high purity graphene nanocarbon products, including carbon nanotubes. Typical SEM, TEM and HAADF (High Angle Annular Dark-Field TEM) elemental analysis imaging of the CNTs are presented in Figure S3, and have been extensively detailed [7].

Control of the CO_2 electrolysis conditions is used to tune the specific GNC generated by control of the temperature, current density, and the composition of the electrolyte [8]. For example, a lower temperature (725°C) is typically used in the electrolytic growth of carbon nano-onions, while higher temperature (750 to 770°C) is used in the electrolytic growth of carbon nanotubes. Lithium carbonate, a typical electrolyte, has a melting point of 723°C. Binary lithium carbonate mixtures have a lower melting point. A high sodium carbonate content in a mixed sodium/lithium carbonate electrolyte and a lower electrolysis temperature (670°C) drive the formation of a graphene scaffold nanocarbon product formation. Applied electrolysis current densities generally range from 0.03 to 0.6 A cm^{-2}. High current density (0.6 A cm^{-2} or over) is

one of the principal conditions driving the formation of fascinating helical, rather than straight, carbon nanotubes.

Electrode (and electrolyte additive) composition variation has been used to grow other GNC allotropes from CO_2. These include carbon nanobamboo, carbon nanopearl, graphene from nanocarbon platelets, carbon nanofiber, carbon nanobelt, carbon nanotree, and other specific carbon allotrope morphologies. SEM of a range of these GNC products is presented in Fig. S4, and XRD and Raman spectra of the products are presented in Figs. S5 and S6 as previously detailed [8].The solid graphene nanocarbon product from CO_2 grows as a matrix directly on the cathode. Under constant current electrolysis conditions, the product formation is continuous, and the growth occurs in the direction towards the anode.

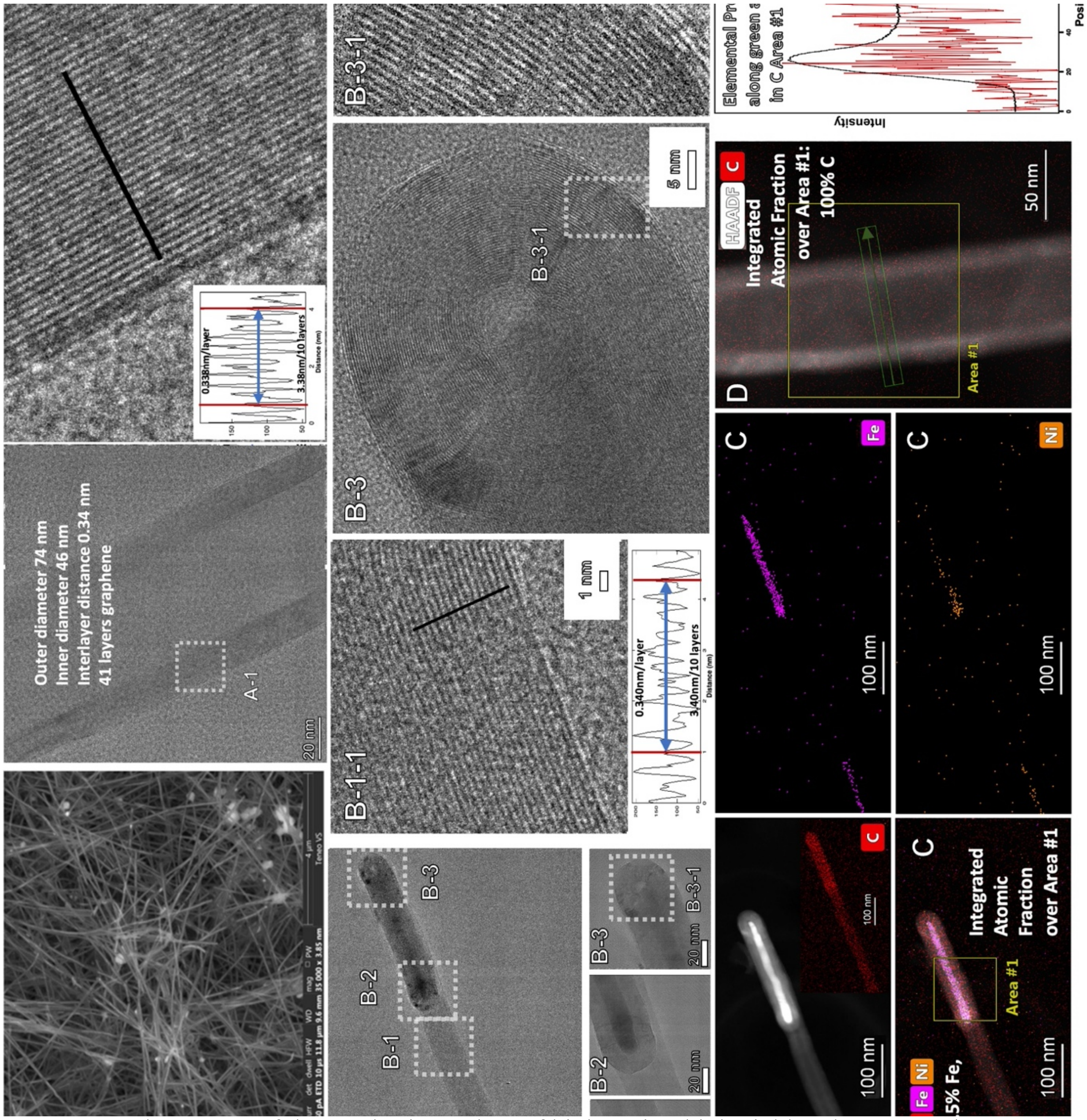

Figure S3. SEM TEM and HAADF of the synthesis product of high purity, high yield carbon nanotubes by electrolytic splitting of CO_2 in 770°C Li_2CO_3. The SEM has a scale bar of 5 µm. Panels B are TEM with scale bars decreasing from 100, 20, 5 and 1 nm. Bottom rows panels C are HAADF elemental analyses with scale bars decreasing from 100 to 50 nm, and in the bottom right a HAADF elemental carbon profile analysis of the carbon nanotube cross section. Modified from open access paper X. Liu, G. Licht, S. Licht, Controlled Transition Metal Nucleated Growth of Carbon Nanotubes by Molten Electrolysis of CO_2 Catalysts 12 (2022) 137. https://doi.org/10.3390/catal12020137.

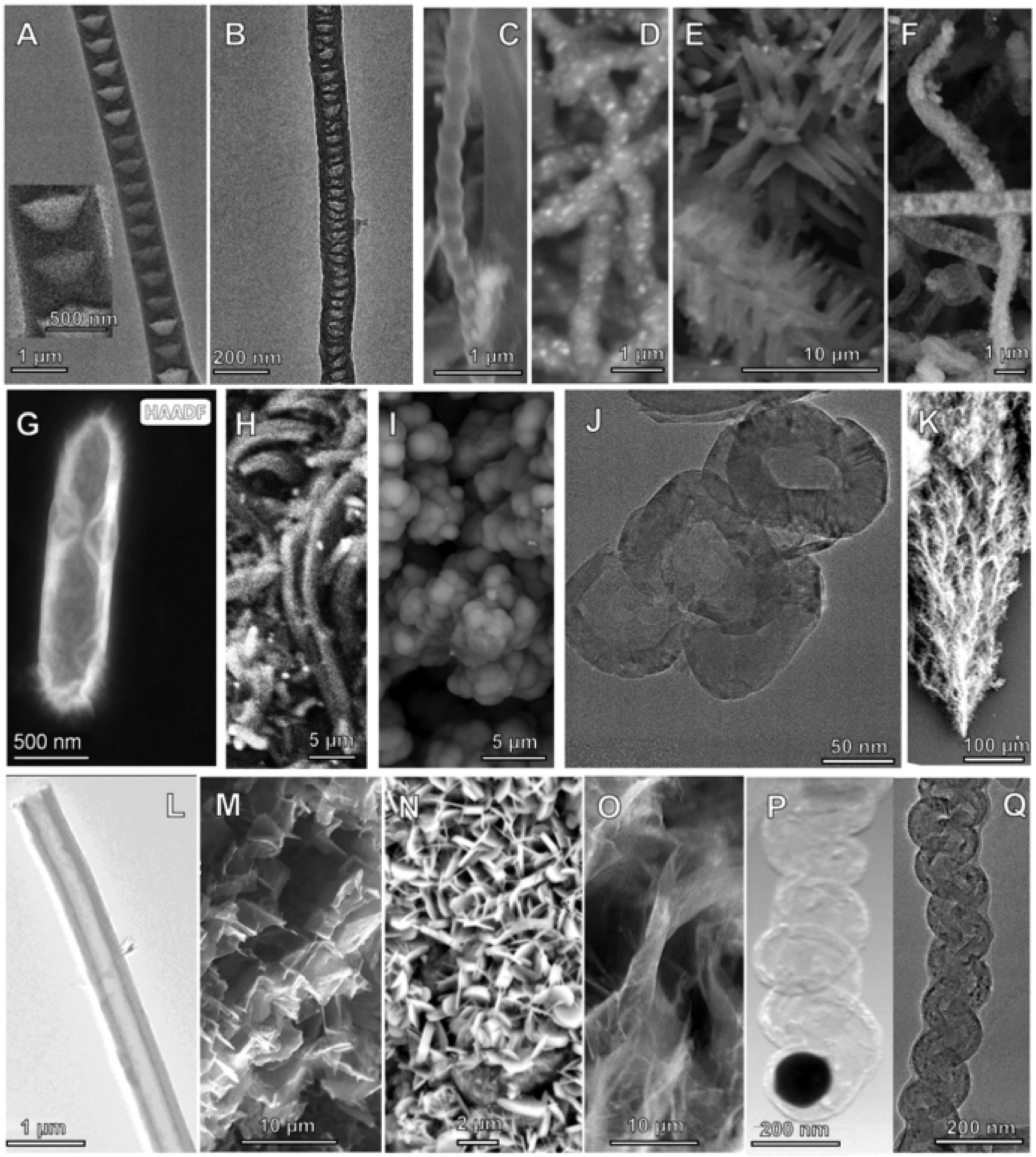

Figure S4. SEM of nanocarbon allotropes synthesized by the electrolytic splitting of CO_2 in molten carbonate. Top row (from A to F) conical CNF, nano-bamboo, nano-pearl, Ni coated CNT, nano-flower, nano-dragon. Middle row (from G to K): nano-rod, nano-belt, nano-onion, hollow nano-onion, and nano-tree. Bottom row (from L to Q) Carbon nanotube, nano-scaffold (ref. 50), nano-platelet, graphene, nano-helices. Modified from open access paper X. Liu, G. Licht, X. Wang, S. Licht, Controlled Growth of Unusual Nanocarbon Allotropes by Molten Electrolysis of CO_2. Catalysts 12 (2022) 137. https://doi.org/10.3390/catal12020125.

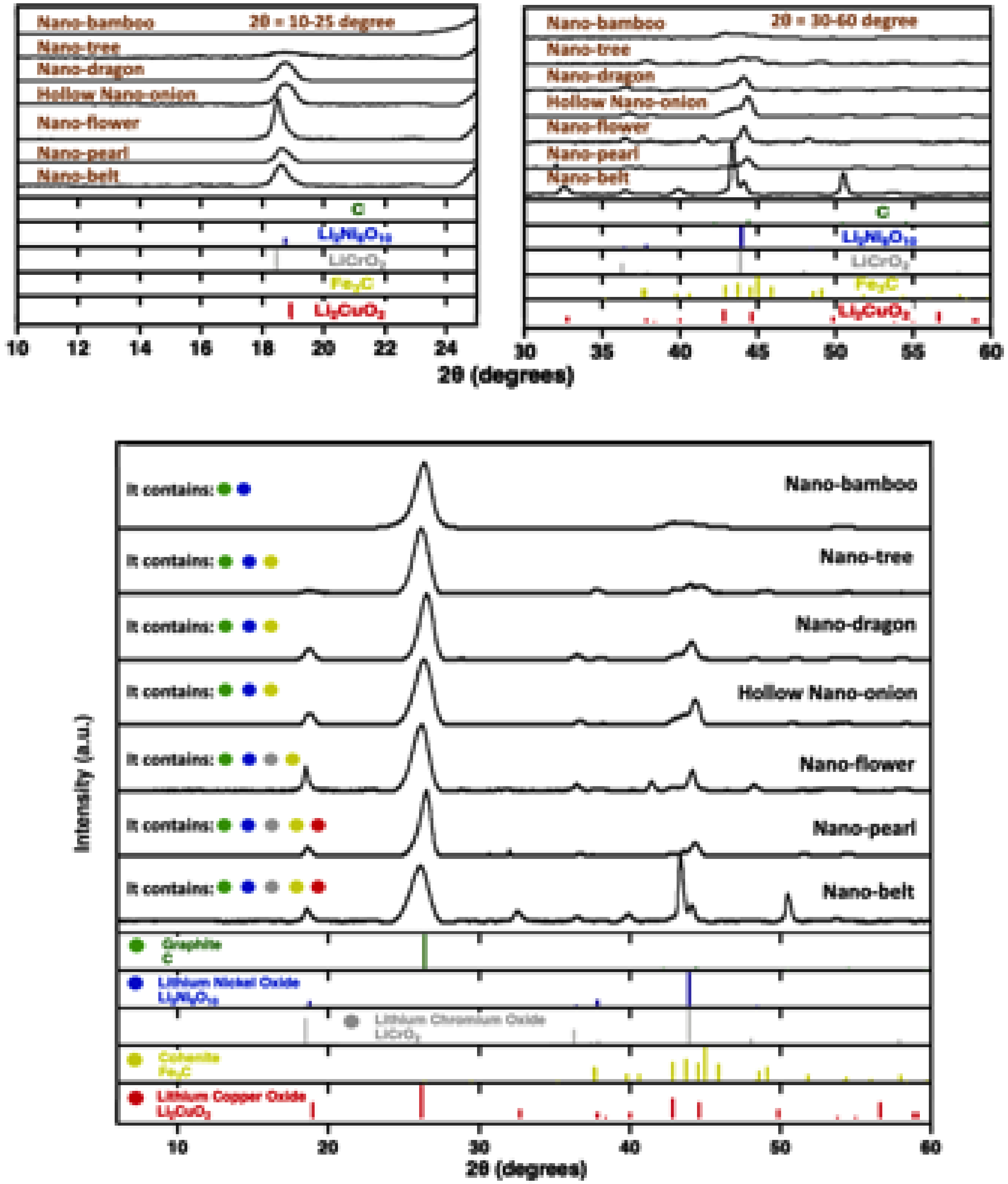

Figure S5. XRD of the synthesis product consisting of various labeled unusual nanocarbon morphologies synthesized by the electrolytic splitting of CO_2 in 770°C Li_2CO_3 with a variety of systematically varied electrochemical conditions. Modified from open access paper X. Liu, G. Licht, X. Wang, S. Licht, Controlled Growth of Unusual Nanocarbon Allotropes by Molten Electrolysis of CO_2. Catalysts 12 (2022) 137. https://doi.org/10.3390/catal12020125.

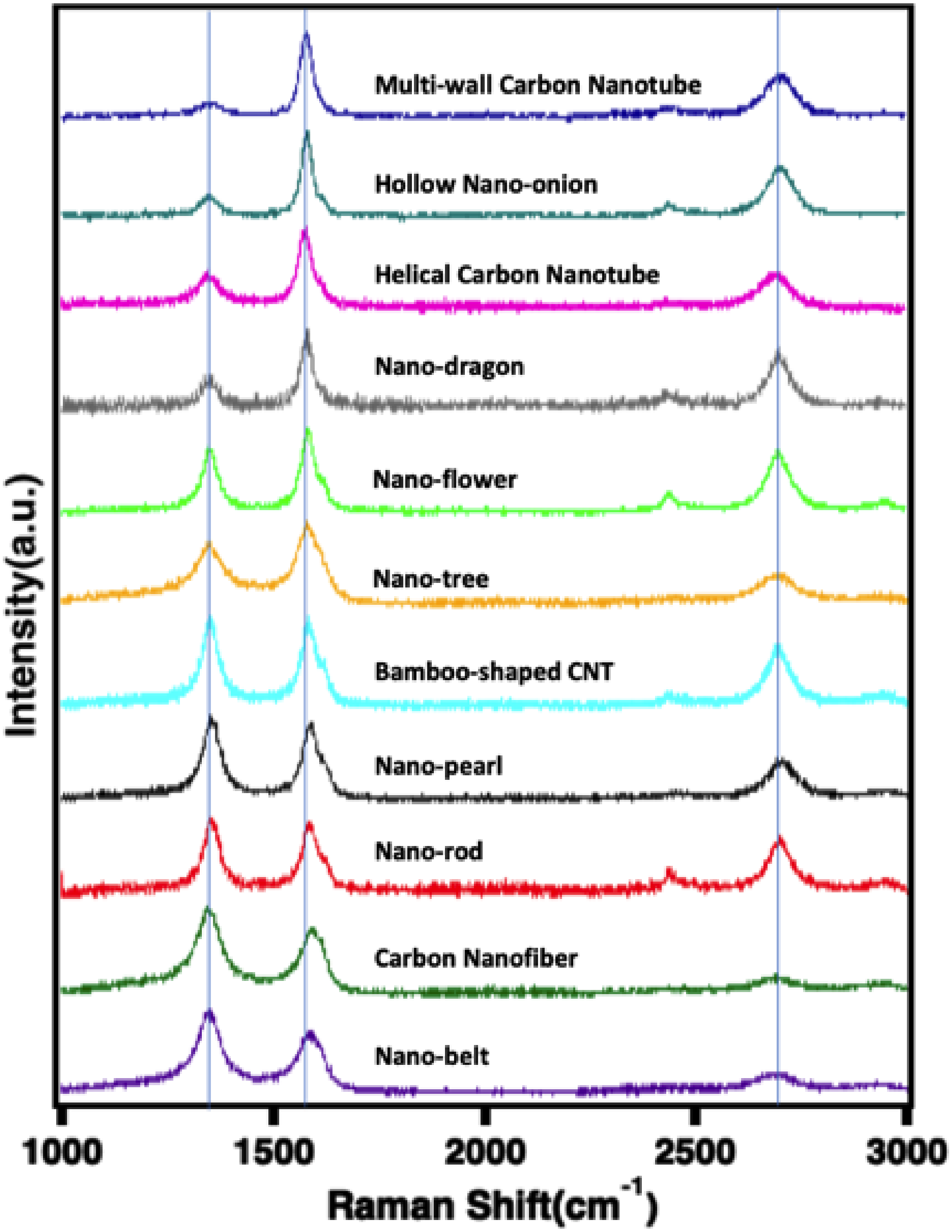

Figure S6. Raman of the synthesis product consists of various labeled GNCs and packed carbon nanotube assemblies synthesized by the electrolytic splitting of CO_2 in 770°C Li_2CO_3 with a variety of systematically varied electrochemical conditions. Modified from open access paper X. Liu, G. Licht, X. Wang, S. Licht, Controlled Growth of Unusual Nanocarbon Allotropes by Molten Electrolysis of CO_2. Catalysts 12 (2022) 137. https://doi.org/10.3390/catal12020125.

References

[1] CO_2-Earth: Daily CO_2 Values. Available online: https://www.CO2.earth/daily-CO2 (accessed on 6 June 2024).

[2] NASA: Global Climate Change: The Relentless Rise of Carbon Dioxide. Available online: https://climate.nasa.gov/climate_ resources/24/ (accessed 6 June 2024).

[3] M. C. Urban, Accelerating extinction risk from climate change, Science 348 (2015) 571–573. https://www.science.org/doi/10.1126/science.aaa4984.

[4] S. L. Pimm, Climate disruption and biodiversity, Curr. Biol. 19 (2009) R595–R601. https://doi.org/10.1016/j.cub.2009.05.055.

[5] G. K. Praksh, G. A. Olah, S. Licht, N. B. Jackson, Reversing Global Warming: Chemical Recycling and Utilization of CO_2; Report of 2008 NSF Workshop; University of Southern California: Los Angeles, CA, USA, 2008. https://www.nsf.gov/mps/che/workshops/2008_ReversingGlobalWarming_NSF_Workshop.pdf (accessed 6 June 2024).

[6] J. Ren, F.-F. Li, J. Lau, L. Gonzalez-Urbina,S. Licht, One-pot synthesis of carbon nanofibers from CO_2. Nano Lett. 15 (2015) 6142–6148. DOI: 10.1021/jp9044644.

[7]X. Liu, G. Licht, S. Licht, Controlled Transition Metal Nucleated Growth of Carbon Nanotubes by Molten Electrolysis of CO_2 Catalysts 12 (2022) 137. https://doi.org/10.3390/catal12020137.

[8] X. Liu, G. Licht, X. Wang, S. Licht, Controlled Growth of Unusual Nanocarbon Allotropes by Molten Electrolysis of CO_2. Catalysts 12 (2022) 137. https://doi.org/10.3390/catal12020125.

[9]G. Licht, K. Hofstetter, S. Licht, Separation of molten electrolyte from the graphene nanocarbon product subsequent to electrolytic CO_2 capture. Decarbon 4 (2024) 100044. https://doi.org/10.1016/j.decarb.2024.100044.
